# Consequences of *Nosema apis* infection for male honey bees and their fertility

**DOI:** 10.1038/srep10565

**Published:** 2015-06-30

**Authors:** Yan Peng, Barbara Baer-Imhoof, A. Harvey Millar, Boris Baer

**Affiliations:** 1 Centre for Integrative Bee Research (CIBER), Bayliss Building M316, The University of Western Australia, Crawley, WA 6009, Australia; 2 ARC Centre of Excellence in Plant Energy Biology, Bayliss Building M316, The University of Western Australia, Crawley, WA 6009, Australia

## Abstract

The queens of eusocial bees, ants and wasps mate only during a very short period early in life and males therefore produce ejaculates consisting of large numbers of high quality sperm. Such extreme selection for high fecundity resulted in males investing minimally into their somatic survival, including their immune system. However, if susceptible males are unable to protect their reproductive tissue from infections, they compromise queen fitness if they transfer pathogens during mating. We used the honey bee *Apis mellifera* and investigated the course of infection of the sexually transmitted pathogen *Nosema apis*. We predicted that honey bee males are susceptible but protect their reproductive tissues from infections. We investigated the effects of *N. apis* infections on the midgut, the accessory glands and the accessory testes and quantified the consequences of infection on male survival and fecundity. We found that *N. apis* is able to infect males, and as infections progressed, it significantly impacted fertility and survival in older males. Even though we confirm males to be able to minimize *N. apis* infections of their reproductive tissues, the parasite is present in ejaculates of older males. Consequently *N. apis* evolved alternative routes to successfully infect ejaculates and get sexually transmitted.

The mating biology of eusocial ants, bees and wasps is truly spectacular because reproductive females (queens) only copulate during a very brief period early in their life when they acquire and store a lifetime’s supply of sperm^1^,^2^,^3^. As queens never replenish sperm later in life, sperm use and economy has been taken to inimitable extremes in those species that maintain colonies with millions of workers^3^,^4^,^5^,^6^ and/or survive in the field for decades^4^,^7^,^8^. To provide queens with sufficient numbers of sperm, social insect males evolved ejaculates that contain large numbers of sperm of exceptionally high quality^3^,^9^. The production and maintenance of such ejaculates prior to mating induces substantial costs to eusocial insect males^5^,^10^. They are therefore thought to operate at their physiological limits, where even the slightest disturbance or alteration in their environment can compromise their fertility^11^. Additionally, to maximise their reproductive potential, these males have been found to only minimally invest into their somatic survival^5^,^10^.

The immunity of social insect males is also affected by their genetics because they originate from non-fertilized eggs and are haploid animals, which has been hypothesized to additionally increase their susceptibility to parasites^12^. Therefore, it is particularly interesting to study immune challenges resulting from parasitic infections in eusocial insect males. Empirical work has confirmed that immune responses of social insect males are consistently lower than those of female workers e.g. in leaf cutter ants^13^, wood ants^14^ or bumblebees^15^. The resulting susceptibility of social insect males to parasitism is thought to have resulted in a number of life history adaptations, which reduce a male’s risk of getting infected: e.g. they have short life spans, are completely dependent on workers and do not provide any help for their colony. However, social insects are known to host a range of parasites, which easily spread through their colonies consisting of large numbers of related individuals including males^16^,^17^. Male susceptibility might therefore reduce a colony’s opportunity to control infections and compromise colony fitness further, especially if such infections reduce male fecundity, life expectancy or competitiveness. Finally, male susceptibility could also allow pathogens to spread through their bodies and eventually infect their reproductive organs. In such a case, sexually transmitted pathogens could then inflict cross-generational costs. However, sexually transmitted diseases and their consequences have not been studied in great detail in social insects^18^,^19^,^20^. Here we hypothesize that social insect males should minimize the risk of spreading a disease to queens by protecting their reproductive tissues from parasite infections.

To test this idea, we used the honey bee *A. mellifera ligustica* and investigated the effects caused by an infection with the widespread fungal disease *Nosema apis*. Honey bees make interesting study organisms to unravel effects of infections on male immunity and fecundity for a number of reasons. First they are known to host a wide variety of parasites^21^,^22^ and second many of these are widespread and abundant enough among colony members to pose a potential threat to males (drones). Additionally, deformed wing virus was successfully identified in honey bee ejaculates, suggesting that some honey bee diseases could be sexually transmitted^19^,^23^. More recently, *N. apis* has been reported to be present in ejaculates of honey bee drones, and artificial insemination experiments confirmed that this pathogen can in principle be transmitted during mating^24^. Consequently, *N. apis* is an ideal study organism to test the effects of parasites on male fertility, and whether male susceptibility allows a disease to spread to both the reproductive organs and the ejaculate. In order to test this, we conducted a series of experiments to unravel the effects of a *N. apis* infection on a male’s somatic tissue, by inspecting infected somatic tissues of drones at different ages and comparing the survival of infected and uninfected drones. We also investigated whether males are able to protect their reproductive organs and their ejaculates from *N. apis* infections to minimize the risk of transmitting infective spores during copulation. To do this we measured the effects of a *N. apis* infection on the drones’ reproductive organs at different ages, and quantified the consequences of such an infection on their fecundity.

## Materials and methods

### *Nosema apis* spore collection

To collect *N. apis* spores, we sampled 20 foraging workers from five different *N. apis* infected honey bee colonies housed at the University of Western Australia and froze all the 100 bees we sampled for 2hrs at -20 °C. We dissected out their midguts, pooled them in an Eppendorf tube containing 1 mL of DDI water and a 3 mm tungsten bead (Qiagen, Australia), and homogenized them in a mixer mill (Retsch MM301, Australia) for 30 s at 25 Hz. Next, we layered 0.5 mL of the homogenate onto 1.5 mL of 100% Percoll (Sigma-Aldrich, Australia) in a 2 mL Eppendorf tube, and centrifuged it at 18,000 x *g* for 60 min at 4 °C, discarding the supernatant. The pellet containing the *N. apis* spores was resuspended using 1.5 mL DDI water. The sample was briefly vortexed and centrifuged again at 20,700 x *g* for 5 min. We repeated this procedure three times before resuspending the final pellet in 0.5 mL of DDI water to store at -80 °C. Prior to infecting drones, we thawed the sample and diluted it with sugar syrup to a final concentration of 10,000 spores/μL. Our previous work showed that collecting *N. apis* spores as described above minimizes the effect on their viability^25^.

### Drone breeding and infection

In summer 2010, we bred drones in two different colonies according to standard apicultural practices by providing each colony with one empty drone comb. Two days prior to hatching, we placed the drone frames into an incubator at 33 °C and 90% humidity and collected drones on the day they emerged (day 0). The first 21 drones collected were used for the following procedures: The faeces of three newly hatched drones was inspected under a microscope for the presence of *N. apis* spores. Next, we freeze-killed 6 newly hatched drones for 2 hours to dissect out their midguts and microscopically check the gut tissue for evidence of a *N. apis* infection. During the dissections we found that their midguts were translucent and detected no signs of a *N. apis* infection, such as spores or morphological changes. In addition, we freeze-killed 6 newly hatched drones to prepare histological samples by embedding their sexual organs (accessory glands and testes) and inspect them for signs of an infection with *N. apis*. Finally, we froze a further 6 newly hatched drones to determine the presence or absence of *N. apis* DNA using microsatellite markers. As we never detected any signs of *N. apis* in any of the 21 newly hatched males inspected (Table 1), we concluded that newly hatched males were not infected with *N. apis*, which is in accordance with the literature^26^.

All remaining eclosing drones were collected and each was fed with 1 μL (10,000 spores) of *N. apis* spore solution (prepared as described above), before we returned them to their maternal colonies. We recaptured 21 drones on each day 6, 9, 13, 15, 20, and 25 days post infection, and used them for the same experiments as described above: 3 drones were immediately checked for spores in their faeces, 6 drones were freeze-killed to dissect and inspect their midguts, 6 drones were used for histological embedding and inspection of their sexual organs, and 6 drones were checked for *N. apis* DNA using microsatellite markers. In addition, to investigate, whether sexually mature drones would be able to transmit *N. apis* spores to the queen during mating, we microscopically inspected ejaculates of 3 infected drones on days 13, 15, 20 and 25 days post infection, respectively. An overview over the sample sizes used for each experiment is provided in Table 1.

### Histological confirmation of *N. apis* spore presence in reproductive tissue

After collecting and freeze-killing 6 drones for each of the 7 age groups as described above, we dissected their reproductive organs (accessory glands and accessory testes). To avoid contamination, we cleaned all equipment after each dissection with ethanol, bleach and water^27^. Furthermore, we rinsed each tissue sample three times prior to histological embedding in Hayes solution (0.15 M NaCl, 1.80 mM CaCl_2_, 2.68 mM KCl, 1.19 mM NaHCO_3_ , adjusted to pH 8.7 using NaOH, filtered (0.22 μm Millex® GP filter unit, America). Next, we transferred each sample into a 2 mL tube and covered it with 1.8 mL cold fixative (2.5% glutaraldehyde, 2% paraformaldehyde) before placing it on a Stovall Belly Dancer ^TM^ Shaker on 10 rpm for 30 min on ice at 4 °C overnight in a dark box. We then transferred the samples into 0.1 M phosphate buffer at pH 7.4, mixing them on ice for 15 min on a Belly Dancer on 10 rpm. Next we placed them into 1% osmium tetroxide (Sigma-Alderich, Australia) for 2 h before rinsing them in phosphate buffer. To dehydrate the tissue samples, we first placed them in an ascending series of ethanol concentrations (50, 70, 90, and 100%) followed by two washes in propylene oxide and embedding into Epon Procure 812-Araldite resin (Polyscience, Inc., Australia), using four steps of increasing concentrations of propylene oxide to resin ratios (1:3, 1:1: 3:1 and pure resin), all according to the manufacturer’s protocol. We finally placed each tissue sample in moulds containing resin at 60 °C overnight. All embedded organs were sectioned with glass knifes using an ultramicrotome (LKB). The sections of 1-2 μm were then de-plasticized using an aged, saturated solution of sodium hydroxide in 100% ethanol (sodium ethoxide) for 5 min. Afterwards, we rinsed them in 100% ethanol and water for 10 s each before placing them in 1% w/v hydrogen peroxide for 7.5 min. Finally, we rinsed each section under tap water for 3 s and stained them with Slidder’s Hematoxylin, Eosin and 1% w/v Biebrich scarlet^28^. Last we mounted the sections on microscopic slides using water-free mounting media (Entellan®, Merkck Millipore, Australia) and investigated them under an Olympus BX51 microscope (Olympus, Japan bright field, Namarski optics with UPLAN FL objective lenses), taking digital photos with an Olympus DP70 camera.

### Detection of *N. apis* in male reproductive tissue using microsatellites

We used specific primers to test for the presence of *N. apis* in the drones’ reproductive organs. To do this we dissected the accessory glands and accessory testes from 6 drones per age group (see Table 1) and rinsed them in Hayes solution. To extract DNA, we placed each sample in 95% ethanol before transferring it into a vial with 500 μL extraction buffer (0.1 M Tris, 0.05 M EDTA, 0.5 M NaCl, 1% w/v Polyvinyl-pyrrolidone) and a 3 mm tungsten bead (Qiagen, Australia). After homogenising each sample for 3 min in a mixer mill at 25 Hz, we added 66 μL of 10% sodium dodecyl sulphate before centrifugation at 20,800 x *g* at 4 °C for 15 min. We added 445 μL of isopropanol to 600 μL of the supernatant and incubated the samples on ice for 15 min before centrifugation at 20,800 x *g* at 4 °C for 15 min. We resuspended the DNA pellet in 500 μL of 70% ethanol and centrifuged each sample again at 20,800 x *g* at 4 °C for 15 min. After discarding the supernatant, each pellet was air dried for 20-45 min before being resuspended in 100 μL of DDI water and centrifuged again at 20,800 x *g* at 4 °C for 15 min. We collected the supernatant and determined the amount of DNA using a NanoDrop (ND-1000 V3.2.1., America). Prior to PCR, we diluted each sample to a final concentration of 50 ng DNA/ μL in DDI water.

We used *N. apis* primer sequences as published in the literature^29^, purchased from Sigma-Alderich, Australia. The forward primer used was 5-GGGGGCATGTCTTTGACGTACTATGTA-3 and the reverse primer was 5-GGGGGGCGTTTAAAATGTGAAACAACTATG-3. We amplified DNA in a S1000™ Thermal Cycler Chassis (Bio Rad, Australia). Each reaction contained 13.4 μL sterile DDI water, 2 μL 10x Taq buffer, 0.1 μL Taq polymerase (Bio-Rad, Australia, Cat# M0267X), 1.0 μL of each forward and reverse primer, 0.2 μL of 10% Triton-x100, 0.5 μL dNTP (Bio-Rad, Australia, Cat# 0447 L), and 2 μL of extracted DNA or sterile DDI water used as a negative control. We denatured the DNA for 5 min at 94 °C (1x cycle); which was followed by 30 reaction cycles consisting of denaturation for 15 s at 94 °C, primer annealing for 30 s at 61.8 °C, extension for 45 s at 72 °C; and a final extension cycle of 7 min at 72 °C (1x cycle).

To confirm the absence or presence of *N. apis* DNA in each sample, we ran 5 μL of the PCR amplification product on 1% agarose gels (Promega, Australia,) in 1x Tris-borate-EDTA buffer with 2% v/v of electrophoresis ethidium bromide (Merck, Australia). We used a 100 bp DNA ladder (Invitrogen, Australia, Cat# 15628-019) as a molecular marker, and a *N. apis* spore sample as a positive control. For gel electrophoresis we used a Bio Rad Mini-Sub® Cell GT Cell (Australia) at 80 V, 400 mA for 30 min. We then photographed the gels in a Bio Rad ChemiDoc™ XRS + System with Image Lab™ Software (Australia) and determined the presence or absence of *N. apis* by checking each sample for species-specific gel bands.

### The effect of *N.apis* infection on drone survival and sperm viability

In 2013, we conducted an additional experiment to quantify the effect of *N. apis* on drone survival and sperm viability at different ages, and bred drones in two different colonies as described above. After hatching, we randomly allocated 20-30 drones to one of 25 cages (14 × 19.5 × 2.3 cm, punctured metal sheet on one side and drone excluder on the other) and placed them back into their maternal colonies for the next 24 h. We recollected the cages and fed the drones with either 1 μL Nosema spore solution (10,000 spores), or with 1 μL of 100% w/v sugar syrup as a control. Consequently we ended up with 12 cages containing infected drones and 14 cages containing uninfected drones. We resampled drones 12, 13, 16, 19, 20, 24 and 25 days post infection. For each cage, we first counted all live and dead drones to quantify drone survival. We anesthetized the surviving drones in chloroform to initiate ejaculation and gently squeezed the drones’ abdomen between two fingers, until semen appeared at the tip of the endophallus^30^. To quantify sperm viability, we used flow cytometry as described by Paynter et al. (2014)^31^. In short, we collected around 2 μL of ejaculate per drone in 1 μL of semen diluent (188.3 mM sodium chloride, 5.6 mM glucose, 574.1 nM arginine, 684.0 nM lysine, 50 mM tris (hydroxymethyl) aminomethane, pH 8.7). We added 1 mL of semen diluent to each semen sample and gently mixed it by turning the vial upside down until the ejaculate had fully dispersed. To avoid mucus clogging up the capillary of the flow-cytometer, we filtered a subsample of 200 μL through a 50 μm diameter nylon mesh and added 800 μL of semen diluent to the filtered sample. To differentially dye live and dead sperm, we used 400 μL of sperm sample, added 2 μL of 1 mM SYBR 14 dye (Invitrogen, cat no. L-7011) and incubated it in the dark for 10 min. We added 2 μL of 2.4 mM Propidium Iodide (PI) (Invitrogen, cat no. L-7011) and incubated the sample for 7 min in the dark. Sperm viability was quantified for a minimum of 3000 sperm in a BD FACS Canto II digital flow cytometer (America). The flow cytometer recorded SYBR 14 fluorescent emission in the range 515–545 nm, and PI fluorescent emission in the range 670–735 nm without compensation for spectral overlap. We recorded height rather than area of the voltage pulse generated by SYBR 14 and PI, because honey bee sperm is exceptionally long (260 μm)^1^. Sperm dyed with SYBR 14 were gated as live and those stained with PI were gated as dead, using the FlowJo software package Version 7.6.5 for Windows (TreeStar, USA). We used the autogate function of the Flowjo software, except in 10 cases where the populations of live and dead sperm could not be separated by the software. As is typically done in these cases we therefore assigned the gates manually. We ignored doubly stained cells, which occurred at very low frequencies. Statistical analyses were performed using SPSS version 21 for Macintosh. Drone ages were grouped for Figures, but statistical analyses were done using drone age as a covariate.

## Results

**Figure 1:**
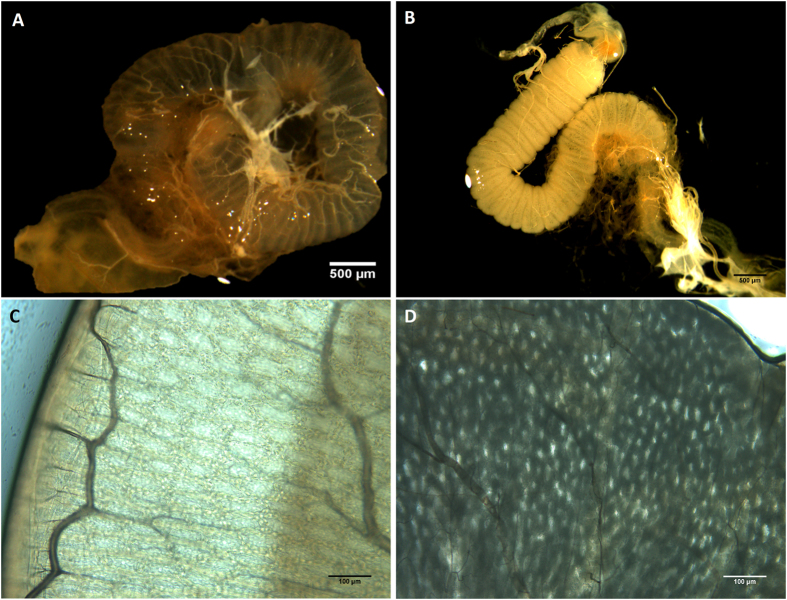
Comparison of the midguts of mature male honeybees that were either uninfected (A = gross morphology and C = magnified) or infected with the microsporidian parasite *Nosema apis* (B = gross morphology and D = magnified).

**Figure 2:**
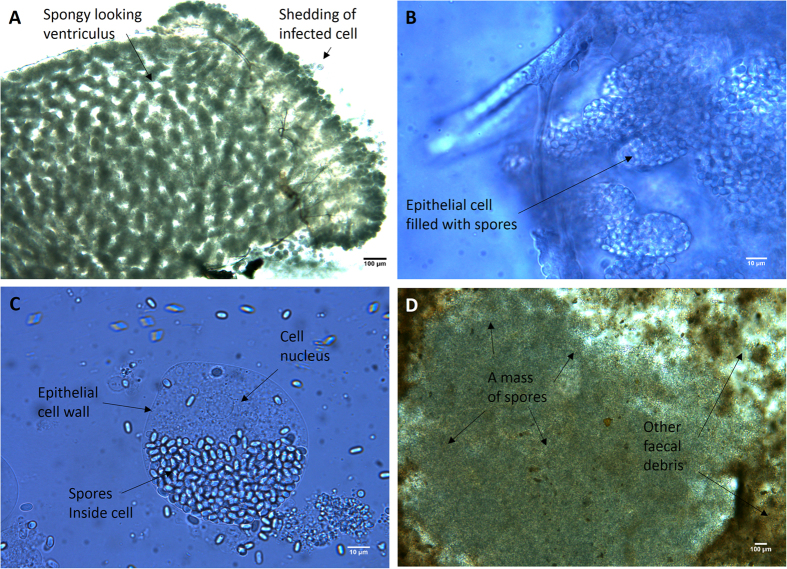
Different views on the midgut and faeces of mature honeybee males infected with the microsporidian parasite *Nosema apis*: A = midgut 20 days post infection, B = magnified view of an infected midgut, C = close-up on the epithelial cells of an infected midgut. D = faeces with pockets of spore masses (within the white border) among other debris (brown).

We found that all drones fed with *N. apis* spores consequently developed an infection (Table 1), which we were able to confirm by the presence of morphological changes that became visible in the midgut of infected compared to non-infected drones (Fig. 1). We found that the midguts of newly hatched, non-infected drones appeared transparent and remained translucent as drones matured (Fig. 1A,C). In contrast, as the infected drones matured, their midguts developed substantial swelling, lost their transparency and changed their colour to a grey-white (Fig. 1B,D). Microscopic inspections confirmed that the epithelial cells of the midguts of infected drones were filled with *N. apis* spores (Fig. 2A-C) and we could observe large numbers of newly-released spores from burst cells (Fig. 2D).

**Figure 3:**
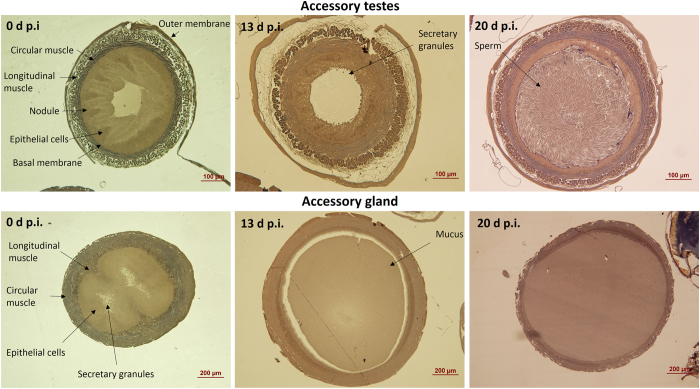
Longitudinal vertical cuts through the accessory testes (top) and accessory gland (bottom) of male honey bees at 0, 13 and 20 days post infection (p.i.) with the microsporidian fungal parasite *Nosema apis*.

**Figure 4:**
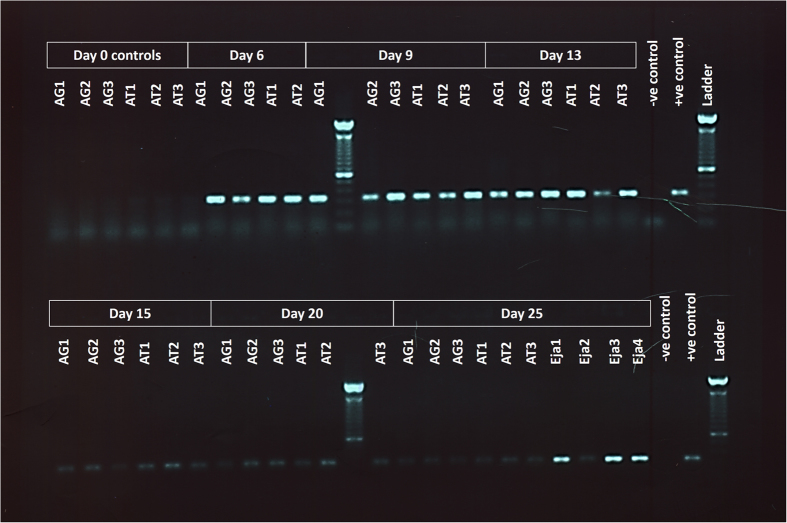
PCR amplification results showing DNA of the microsporidian parasite *Nosema apis* in the reproductive tissues of infected, mature male honey bees. Abbreviations: AG = accessory glands, AT = accessory testes, Eja = ejaculate, -ve control = water used as negative control,+ve control = samples known to contain *Nosema apis* DNA used as a positive control.

**Figure 5:**
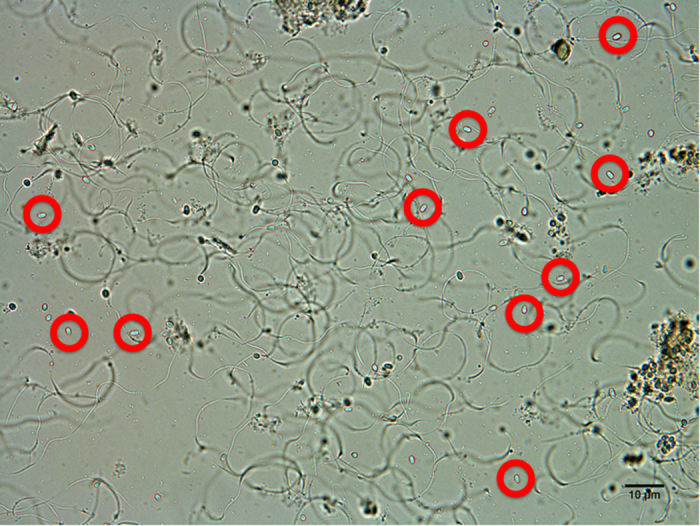
Self-ejaculated semen of a 20-day old honey bee male infected with the microsporidian parasite *Nosema apis*, showing sperm and mucus particles, as well as reproductive spores of the parasite.

When we compared dissected accessory glands and accessory testes of infected and non-infected drones (Fig. 3) we did not find any of the morphological signs of infection as described above for the midgut tissue (Fig. 1 & 2). Furthermore, we never found any *N. apis* spores to be present in the sexual organs of infected drones of any age, neither within the tissue nor in the lumen containing either sperm or seminal fluid. This observation was confirmed histologically when comparing the sexual tracts of non-infected and infected drones of different ages. We found that the muscular tissue surrounding the accessory gland and its epithelial cell layer gradually degenerated with increasing drone age (Fig. 3), which was not the case for the muscular tissue surrounding the accessory testes (Fig. 3). However, neither the accessory glands nor the accessory testes tissues showed any visible signs of infection (Fig. 3). Furthermore, despite a very careful microscopic inspection of both the accessory testes and accessory glands, we never found a single *N. apis* spore in any of the infected males. However, our PCR analysis detected *N. apis* DNA in male reproductive tissues (Fig. 4). This was the case for all drone age groups except for the newly emerged drones, confirming that newly eclosed drones are not infected with *N. apis*. Finally, when we microscopically inspected ejaculated semen, *N. apis* spores were only present in drones aged 20 and 25 days (Fig. 5), but never in younger drones at 9, 13 or 15 days of age.

### Effect of *N. apis* infection on drone fecundity and survival

**Figure 6:**
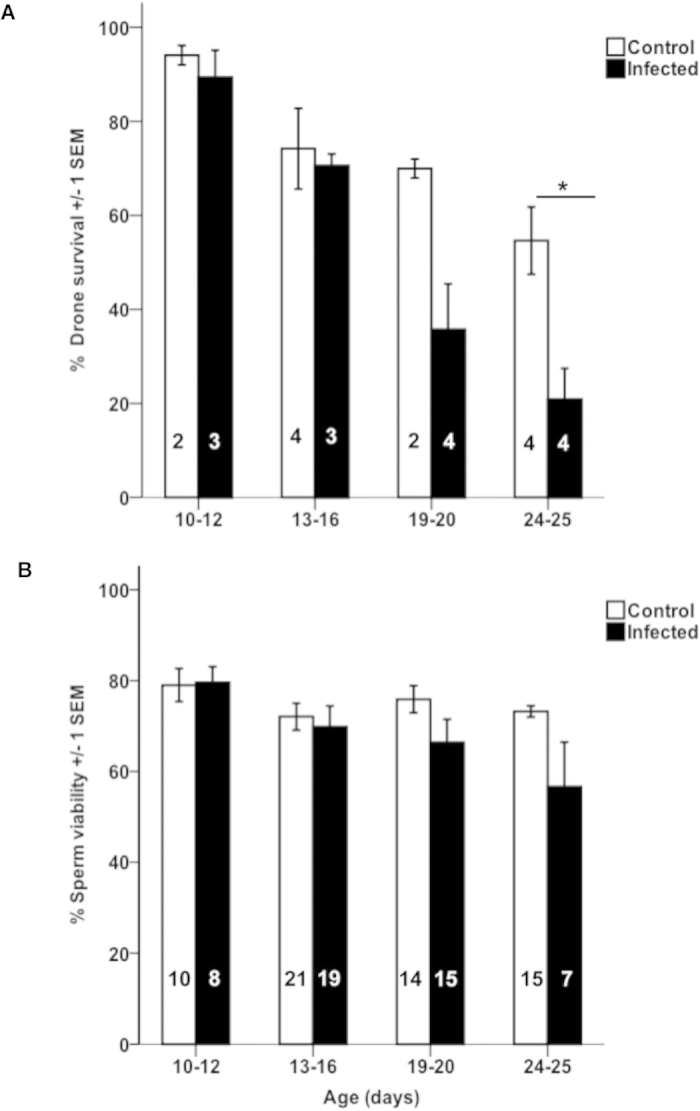
Comparison of honey bee males in different age groups that were either fed with spores of *Nosema apis* (Infected), or sugar syrup (Control): **A**) Percentage of drone survival per cage, numbers in bars refer to the number of cages used for each age group (20-30 bees per cage) **B**) Percentage of sperm viability per ejaculate. Numbers within bars indicate the number of drones used for each age group.

Sperm viability data became available for 49 infected and 60 non-infected males. We found that drone survival significantly decreased with increasing age (ANCOVA, P < 0.001, see Table 2 for statistical details, Fig. 6 A).There was a significant effect of *N. apis* infection on male mortality, indicated by a significant treatment x age interaction (ANCOVA, df = 1, P = 0.013, Fig. 6 A). Drone survival was similar between infected and non-infected drones until they reached an age of about 16 days, after which mortality in infected drones substantially increased compared to the control treatment. Pairwise post-hoc t-tests using age groups revealed that a significantly lower proportion of treated drones (20.7%) per cage survived to the age of 24 to 25 days compared to control drones (54.7%), with t = 3.5, df = 6 and p = 0.013. The results of the post-hoc tests for the other age groups were non-significant.

Similarly, sperm viability also decreased with male age (ANCOVA, P = 0.003, see Table 3 for statistical details, Fig. 6 B). We also found a significant treatment x age interaction term, indicating that infected males lost their fertility faster than control drones (ANCOVA, P = 0.013, see Table 3). The results of individual, pairwise post-hoc t-tests using age groups were not significant.

## Discussion

Our experiments revealed that *N. apis* is able to infect drones, and that these infections built up to a point where they induced significant costs for males. We found a reduction in fertility and life span as drones aged (Fig. 6), as well as ejaculates of infected drones becoming contaminated with spores (Fig. 4). Honey bee drones become sexually mature from 12 days after emergence and consequently maintain their maximal fertility potential over a time period of approximately 10 days^32^. During that time, they participate in nuptial flights in order to find and mate with virgin queens. The phenotypic expression of a *N. apis* infection such as spores in the ejaculate and a reduction of drone fertility and survival therefore affects these drones during their main reproductive period. These findings indicate that drones which become infected shortly after hatching will eventually face substantial fitness costs and pose an infection threat to virgin queens in case they mate. It would therefore be interesting to investigate, whether infected drones indeed leave their colonies for nuptial flights and if so, whether their mating success is compromised compared to that of non-infected males. From a colony’s perspective, infected males might choose not to participate in mating flights and mating in order not to compromise the mating success of their non-infected brothers. Such “altruistic” self-removal has been reported for honey bee workers after prolonged CO_2_ narcosis or fed with the cytostatic drug hydroxyurea. Both treatments increased worker-mortality, and surviving foragers left their colonies, effectively committing altruistic suicide^33^. Such self-removal is also known from other social insects^34^. More research is required to quantify the risk of vertical transmission posed by males infected with *N. apis*.

Although our visual inspections, both morphologically as well as histologically, did not reveal any obvious signs of *N. apis* infections in reproductive tissues or their products, DNA of *N. apis* can be detected in both accessory glands and accessory testes. These findings indicate that *N. apis* is able to establish low levels of infections in reproductive tissues of drones. However, as we did not find spores in any of the reproductive tissues we inspected, the pathogen seems unable to complete its reproductive cycle or to build up an infection. Therefore, even though *N. apis* is able to infect drone accessory testes and accessory glands, the drones seem able to slow down or prevent this parasite from producing spores within their reproductive tissue, thereby reducing the risk of sexual transmission. This is an interesting finding, because *N. apis* infections are already known to spread to different honey bee organs such as the fat body, the malpigian tubules or the haemolymph^35^. In comparison, *Nosema ceranae* has been reported to infect drones at the pupal stage already^36^, to reduce drone body weight and life span and to induce physiological changes in honey bee queens^37^. Even though drones are more susceptible to *N. ceranae* than workers, they were are to develop higher tolerance to *N. ceranae* in specifically selected honey bee strains^38^. It would therefore be interesting to study how males are able to protect their reproductive tissue, and whether the costs associated with suppressing *N. apis* infections result in the observed decrease in sperm viability and male survival.

We did find *N. apis* spores in the semen of older drones, which raises the question of how they were able to contaminate the ejaculate. Our dissection work revealed that *N. apis* inflicts substantial damage to tissues such as the midgut, which becomes increasingly more fragile with age and can easily be damaged. Furthermore, *N. apis* is known to cause dysentery in honey bees, resulting in spore-containing faecal residues on bees^39^,^40^. It is therefore possible that the spores we detected in the ejaculate of older drones are either caused by faecal contaminations of the endophallus or haemorrhaging after tissue damage. Ejaculation is a traumatic process as the drone contracts its abdominal muscles in order to build up haemolymph-pressure, inducing the irreversible expulsion of the endophallus and then the ejaculate. During a final step of ejaculation, the tip of the drones’ endophallus bursts, breaks off and is left inside the queen after copulation^41^, causing the drones’ death. It therefore seems reasonable to assume that infected tissues, which are more prone to damage and tear already, release *N. apis* spores under the pressure build-up described above, resulting in spore contamination of the ejaculate. Further experimental work is required to unravel these proximate mechanisms. However, if infections occur during the ejaculation process, a male’s only counter measure to protect his ejaculate and mate would need to be derived from immune traits present within the ejaculate. Interestingly, drone ejaculates not only consist of sperm, but also of substantial amounts of seminal fluid, produced by the accessory glands^1^,^42^. The latter is biochemically complex and contains a number of immune proteins, some of them with well-known antifungal properties such as chitinase^43^. Further research should therefore test the idea that seminal fluid is able to kill *N. apis* spores, in order to minimize the risk of *N. apis* establishing an infection inside the queen.

## Additional Information

**How to cite this article**: Peng, Y. *et al*. Consequences of Nosema apis infection for male honey bees and their fertility. *Sci. Rep.* **5**, 10565; doi: 10.1038/srep10565 (2015).

## Figures and Tables

**Table 1:** Number of honey bee males used to investigate the prevalence of *Nosema apis* in faeces, midgut, sexual organs and ejaculates of drones of 7 different ages.

*Nosema apis* prevalence in		Drone age							
		0	6	9	13	15	20	25	Total drones checked
							
							
**Faeces**		0 (3)	0 (3)	3 (3)	3 (3)	3 (3)	3 (3)	3 (3)	21
**Midgut**		0 (6)	6 (6)	6 (6)	6 (6)	6 (6)	6 (6)	6 (6)	42
**Sexual Organs**	Histology	0 (6)	0 (6)	0 (6)	0 (6)	0 (6)	0 (6)	0 (6)	42
	Microsatellites	0 (6)	6 (6)	6 (6)	6 (6)	6 (6)	6 (6)	6 (6)	42
**Ejaculates**		†	†	†	0 (3)	0 (3)	3 (3)	3 (3)	12
**Drones checked per age group**		21	21	21	24	24	24	24	159

Figures provide cases in which *N. apis* was successfully detected, numbers in brackets provide the total number of males inspected. †These males were not sexually mature and therefore no ejaculates could be collected.

**Table 2:** Survival of uninfected honey bee males and males infected with *Nosema apis.*

Source	Type III Sum of Squares	df	Mean Square	F	Sig
**Corrected Model**	**14503.922**^**a**^	**3**	**4834.641**	**27.909**	**<0.001**
**Intercept**	**31752.534**	**1**	**31752.534**	**183.301**	**<0.001**
**Treatment**	**438.758**	**1**	**438.758**	**2.533**	**0.126**
**Age**	**10205.249**	**1**	**10205.249**	**58.913**	**<0.001**
Treatment * Age	**1278.118**	**1**	**1278.118**	**7.378**	**0.013**
**Error**	**3637.757**	**21**	**173.227**		
**Total**	**105605.196**	**25**			
**Corrected Total**	**18141.680**	**24**			

Results from an analysis of covariance examining the effects of an infection with the microsporidian parasite *Nosema apis* on the survival of male honey bees at different ages. Dependent variable: % of drone survival per cage. In a first analysis we used treatment as fixed factor, colony as random factor and age as a covariate. Because colony was non significant (F = 0.210, df = 1, P = 0.727), we consequently removed colony as a factor for the analysis shown. a. R Squared = .799 (Adjusted R Squared = .771).

**Table 3:** Sperm viability of uninfected honey bee males and males infected with *Nosema apis*.

Source	Type III Sum of Squares	df	Mean Square	F	Sig
**Corrected Model**	**3829.692**^**a**^	**3**	**1276.564**	**5.664**	**0.001**
**Intercept**	**51845.693**	**1**	**51845.693**	**230.047**	**>0.001**
**Treatment**	**793.490**	**1**	**793.490**	**3.521**	**0.063**
**Age**	**2119.431**	**1**	**2119.431**	**9.404**	**0.003**
Treatment * Age	**1427.795**	**1**	**1427.795**	**6.335**	**0.013**
**Error**	**23663.822**	**105**	**173.227**		
**Total**	**588393.693**	**109**			
**Corrected Total**	**27493.514**	**108**			

Results from an analysis of covariance examining the effects of an infection with the microsporidian parasite *Nosema apis* on the sperm viability of male honey bees at different ages. Dependent variable: % live sperm per ejaculate counted. In a first analysis we used treatment as fixed factor, colony as random factor and age as a covariate. As colony was non significant (F = 2.290, df = 1, P = 0.381) we removed it for the final analysis.A. R Squared = .139 (Adjusted R Squared = .115).
